# A Chemically Safe Way to Produce Insect Biomass for Possible Application in Feed and Food Production

**DOI:** 10.3390/ijerph17062121

**Published:** 2020-03-23

**Authors:** Cristina Truzzi, Anna Annibaldi, Federico Girolametti, Leonardo Giovannini, Paola Riolo, Sara Ruschioni, Ike Olivotto, Silvia Illuminati

**Affiliations:** 1 Dipartimento di Scienze della Vita e dell’Ambiente, Università Politecnica delle Marche, via Brecce Bianche, 60131 Ancona, Italy; f.girolametti@pm.univpm.it (F.G.); s1084792@studenti.univpm.it (L.G.); i.olivotto@univpm.it (I.O.); s.illuminati@univpm.it (S.I.); 2 Dipartimento di Scienze Agrarie, Alimentari ed Ambientali, Università Politecnica delle Marche, 60131 Ancona, Italy; p.riolo@univpm.it (P.R.); s.ruschioni@univpm.it (S.R.)

**Keywords:** *Hermetia illucens* prepupae, black soldier fly, coffee silverskin, microalgae, potentially toxic elements, bioaccumulation, chemical hazard

## Abstract

Black soldier fly (*Hermetia illucens*, HI, Diptera, Stratiomydae) has great potential as a food and feed ingredient in the European Union (EU). The production of insects as livestock feed or as food ingredients requires strict monitoring of the content of potentially toxic elements (PTEs) in the growth substrate, to meet the security requirements. This study aims to investigate the presence of PTEs, like cadmium, lead, mercury, arsenic, and nickel, in HI prepupae and in their growth substrates based on coffee roasting by-product and microalgae *Schizochytrium* sp. and *Isochrysis* sp. Analyses were carried out via graphite furnace atomic absorption spectrophotometry for Cd, Pb, Ni, and As, and via Direct Mercury Analyzer for Hg. All element concentrations found in growth substrates were below the legal limit of undesirable substances in animal feed (2002/32/EC). Elements concentrations in HI prepupae were in the range (mg kg^−1^ wet weight) of 0.072 to 0.084 for Cd, 0.018 to 0.026 for Pb, 0.010 to 0.032 for Hg, 0.036 to 0.047 for As, and 0.18 to 0.76 for Ni. Even if HI prepupae accumulated Cd, Pb, and Hg, our results indicated that the risk of exposure to PTEs from HI prepupae consumption is relatively low and in compliance with EU regulations.

## 1. Introduction

In light of the predicted increase in the world population by 2050 and the growing demand for high-quality protein sources for food and feed production, insect culture deserves special attention [1,2,3]. In fact, insects show a high protein and fat content, they can grow on organic by-products, their rearing is characterized by low environmental impact [4,5,6] as they produce low greenhouse gases and ammonia emissions [1,7,8], and they show low water and space requirements [5]. Among other insects, the black soldier fly (*Hermetia illucens*, HI Diptera, Stratiomydae) has been proposed by the European Food Safety Authority Scientific Committee [9] as one of the species with the greatest potential as food and feed ingredient in the European Union (EU). HI larvae are characterized by a high protein (up to 42%) and fat (up to 30%) content [10,11,12], a short life cycle [13], low environmental footprint [14], and preference for organic by-products as growth substrate [4,15,16]. Therefore, HI is one of the most promising insect species to meet the future lack of conventional feed and food ingredients, the excessive production of agro-food waste [17,18,19], and the mitigation of climate change [20].

Additionally, it should be underlined that the organic by-products used as feed for the insects can be often contaminated by pollutants. Among several pollutants, potentially toxic elements (PTEs) deserve special attention because of their high degree of toxicity and their wide distribution in the environment [21,22,23]. Most of them, such as arsenic (As), cadmium (Cd), lead (Pb), and mercury (Hg) rank among the priority elements that are of great public health significance. They are all systemic toxicants that are known to induce multiple organ damage, even at lower levels of exposure [21]. PTEs eventually present in growth substrates can be transferred to the insect larvae, and therefore enter the feed/food chain. It has been demonstrated that larvae of mealworm (*Tenebrio molitor*, Coleoptera Tenebrionidae) reared on olive fruits processing by-products can accumulate Pb and Hg [24], whereas Biancarosa et al. (2018) [25] evidenced that HI grown on seaweed-enriched media accumulated significant amounts of PTEs such as Cd, Pb, and Hg. However, knowledge on chemical hazards associated to insects as potential ingredients for feed and food is scarce [9], but the interest about this topic is increasing [2,3,6,12,26,27]. Most studies used artificially-contaminated growth substrates to investigate the potential accumulation of PTEs in insects [28,29,30,31], and specifically in HI [20,32,33,34]. Therefore, to meet a safe production of insects, as well as the need to look for other substrates compatible with European rules and safe about chemical contaminants, a strict monitoring of PTEs content is necessary [20,26]. The EU regulation 2017/893 [35], aside from identifying the insect species that can be cultured, poses some limitations on the substrates that can be used to grow insects (Annex X).

In a previous study performed by our research group, we developed a valid method to improve the polyunsaturated fatty acids (PUFAs) content of HI prepupae by adding to the main growth substrate (coffee silverskin: a coffee roasting by-product) microalgae such as *Schizochytrium* sp. or *Isochrysis* sp. [36]. In this sense, the lack of PUFAs in the insect biomass was bypassed, satisfying at the meantime the EU regulation 2017/893 [35] that imposes the use of “products of non-animal origin” for culturing insects intended for feed production. However, in the light of feed and food security and safety, investigating the possible chemical hazards of each insect feed ingredient should be a priority.

Therefore, the present study aims to investigate the presence of PTEs, such as Cd, Pb, As, Ni, and Hg, according to EU Regulations [37,38]. Specifically, analyses have been carried out on the single growth substrate ingredients (coffee silverskin, *Schizochytrium* sp., and *Isochrysis* sp.), on growth substrates, on HI prepupae reared on these substrates and on frass (excrement from larvae mixed with substrate residues and exuviae). Bioaccumulation of contaminants occurring during insect culture has been evaluated in order to better define the safety traits of the final insect biomass produced.

## 2. Materials and Methods

### 2.1. Insect Growth Substrate Preparation 

Nine different growth substrates were tested during the experiment. The basal substrate consisted of by-products obtained from roasting coffee (a mixture of Arabica and Robusta varieties) process (coffee silverskin, CS), provided by Saccaria Caffè S.R.L. (Marina di Montemarciano AN, Italy). CS (moisture 44%) was collected in plastic bags, frozen at −20 °C, and ground in an Ariete 1769 food processor (De’Longhi Appliances Srl, Italy) to a particle size of 2 ± 0.4mm before the growth substrate preparation. Freeze-dried *Schizochytrium* sp. and *Isochrysis* sp. were provided by AlghItaly Società Agricola S.R.L. (Sommacampagna, VR, Italy) and stored at 4 °C. Growth substrates were formulated as follows; control substrate E, 100% coffe silverskin (CS); substrates As, Bs, Cs, and Ds: CS added with 5%, 10%, 20%, and 25% of *Schyzochytrium* sp., respectively; substrates Ai, Bi, Ci, and Di: CS added with 5%, 10%, 20%, and 25% *Isochrysis* sp., respectively. Water was added to all the substrates, to reach an optimal moisture close to 70% [10], as reported in Truzzi et al. (2020) [36]. Microalgae and growth substrates samples were stored at −20 °C for PTEs determination.

### 2.2. Rearing of Hermetia Illucens Larvae

HI rearing was carried out at the D3A experimental facility (Polytechnic University of Marche) starting from 6-day-old larvae purchased from Smart Bugs s.s. (Ponzano Veneto, TV, Italy). Larvae were divided in the following groups (five replicates per group; each containing 150 larvae) [39]; HI E, prepupae reared on control substrate E (100% CS); HI As, HI Bs, HI Cs, and HI Ds: prepupae reared on substrate CS enriched with 5%, 10%, 20%, and 25% (W/W) of *Schyzochytrium* sp, respectively; HI Ai, HI Bi, HI Ci, and HI Di: prepupae reared on substrate CS enriched with 5%, 10%, 20%, and 25% (W/W) of *Isochrysis* sp., respectively. Groups contained 750 larvae (6 days old, hand counted). Rearing conditions are explained in detail in Truzzi et al. (2020) [36]. Briefly, larvae were reared in a climatic chamber at 27 ± 1 °C and 650 ± 50 g/kg relative humidity [40] in continuous darkness. Each larva was provided with a feeding rate of 100 mg/day [41] within plastic boxes (28 × 19 × 14 cm) for about 1 month (from 6-day-old larva to prepupa stage). Larvae were visually inspected every day and when prepupae were identified by the change in tegument color from white to black [42], they were manually collected using forceps and brushes, immediately frozen, and stored at −20 °C for further analyses. Approximately 10 g of single ingredients and growth substrates were stored at −20 °C for further analyses. At the end of the experiment, frasses were also collected and stored at −20 °C. Experiments were performed in compliance with the Italian laws and institutional guidelines. No specific authorization is requested to conduct experiments on invertebrates such as insects.

### 2.3. Laboratory and Apparatus

A clean room laboratory ISO 14644–1 Class 6, with areas at ISO Class 5 under laminar flow, was used for all laboratory activities. Samples were handled with plastic materials (low-density polyethylene 30 mL cylindrical containers, Kartell, Milan, Italy, Mod K912), washed with acid-cleaning procedures, and rinsed with Milli-Q water obtained from a two-stage system Midi (Elix and Milli-Q) from Millipore (Bedford, MA, USA), to avoid any sample contamination [43]. The laboratory analytical balance was the AT261 Mettler Toledo (Greifensee, Switzerland, readability 0.01 mg, repeatability SD = 0.015 mg). Variable volume micropipettes and neutral tips were from Brand (Wertheim, Germany, Transferpette).

### 2.4. Chemical Analyses and Quality Control

Samples of single ingredients (CS and microalgae), growth substrates, HI prepupae, and frasses were minced, homogenized (homogenizer MZ 4110, DCG Eltronic), and divided in aliquots of 0.5 g each. To determine the moisture, samples were accurately weighed with the analytical balance AT261 (Mettler Toledo, Greifensee, Switzerland) and freeze-dried (Edwards EF4 modulyo, Crawley, Sussex, England) until constant weight (±0.2 mg). Analyses were carried out on three aliquots per sample. For the determination of Cd, Pb, Ni, and As, samples were digested in a high-quality (65% w/v) nitric acid HNO_3_ and 30% v/v H_2_O_2_ (Merk) mixture in a Microwave-Accelerated Reaction System, MARS-X, 1500 W (CEM, Mathews, NC, USA) and the operational parameters were as in Truzzi et al. (2019) [24].

Quantitative determinations of Cd, Pb, Ni, and As were made with an Agilent DUO 240FS atomic absorption spectrometer (Agilent, Santa Clara, CA 95051, USA) equipped with a graphite furnace (GTA120 Graphite Tube Atomizer) and a Zeeman-effect background corrector. The analytical methodology and instrumental parameters were described earlier [24]. Atomic absorption spectrometry standard solutions for Cd, Pb, As, and Ni (Titrisol grades from Merk) were used to build up the calibration curves. Procedural blanks accounted for less than 1% of the total element concentrations in samples.

The total mercury content was quantified by thermal decomposition amalgamation atomic absorption spectrometry (TDA AAS) [44] using a Direct Mercury Analyzer (DMA-1, Milestone, Sorisole, BG, Italy). The homogenized samples were weighed directly into quartz containers. The optimized reading conditions for mercury determination in feed and in insects were as in Truzzi et al. (2019) [24]. It was not possible to perform frass analysis with DMA-1, as the catalytic tube was rapidly destroyed during the analysis. Calibration curve technique was used for the quantification of mercury content [45]. To correct for possible mercury contamination during the analysis, the mercury concentration of a blank was subtracted from sample Hg concentrations.

All analyses were carried out in triplicate. Analytical quality control was achieved using the certified reference material: DORM-2 Dogfish muscle (National Research Council of Canada). Table 1 shows the validation parameters for the analytical procedures. Results were in good agreement with the certified values, and the standard deviation was low, proving good repeatability of the methods.

### 2.5. Bioaccumulation Factor

The bioaccumulation factor (BAF) was calculated on a dry weight (dw) basis [34], as the ratio element concentration in the organism/element concentration in the feed provided. Thus, a BAF greater than 1 suggests bioaccumulation of the element from the substrate into the insect.

### 2.6. Statistical Analysis

Data are expressed as mean ± standard deviation (SD) of the performed replications. After testing the homogeneity of variance with Levene’s test, we verified the normal distribution of data, therefore, to evaluate significant differences among different substrates (at the 95% confidence level), data were subjected to the one-way analysis of variance (ANOVA), followed by the Multiple Range Test [46]. When the ANOVA test gave a *p*-value equal to 0.0000, in the text it was indicated as *p* < 0.0001. All statistical treatments were performed using STATGRAPHICS 18 Centurion [47].

## 3. Results and Discussion

### 3.1. Potentially Toxic Elements Content in Growth Substrate Ingredients

PTEs content in the ingredients used to prepare the growth substrates was reported in Table 2. In coffee silverskin, the concentrations of Cd, Pb, As, and Hg were found to be very low, i.e., less than 0.15 mg kg-^1^ dw. Ni showed the highest content, with 3.5 ± 0.2 mg kg^−1^ dw. Cd, Pb, As, and Ni concentrations are consistent with literature data or even lower [48,49,50,51], whereas Hg showed a 3-fold higher content than the one reported by Zarrinbakhsh et al. (2016) [51], but of the same order of magnitude. It is known that coffee, such as other plant species used to prepare stimulant beverages, contains PTEs, which are present in different concentrations depending on several factors, such as soil profile, plant genetics, and meteorological conditions [48]. CS showed a PTEs concentration in agreement with that normally found in plant used to prepare stimulant beverages, confirming that CS did not present chemical hazard and can be safely used as ingredient to prepare growth substrates for HI rearing.

The content of PTEs in freeze-dried microalgae *Schizochytrium* sp. and *Isochrysis* sp. ranged from micrograms and tens of micrograms per kg (dw) for Cd, Hg, and Pb, to hundreds of micrograms per kg (dw) for As, whereas Ni showed the highest concentration between indagated PTEs. As these microalgae were reared by a company that produces food products, we expected to find low values of these PTEs. However, it should be pointed out that no data about PTEs content in tested microalgae are available in the literature. When comparing coffee silverskin to the two microalgae species it was evident that (i) both microalgae showed significantly lower Cd and Hg concentrations, and a significantly higher Pb content with respect to CS; (ii) *Schizochytrium* sp. showed a significantly higher As content with respect to CS and *Isochcysis* sp; and (iii) *Isochcysis* sp. showed a significantly lower Ni content and a significantly higher content of Pb with respect to CS and *Schizochytrium* sp.

**Figure 1:**
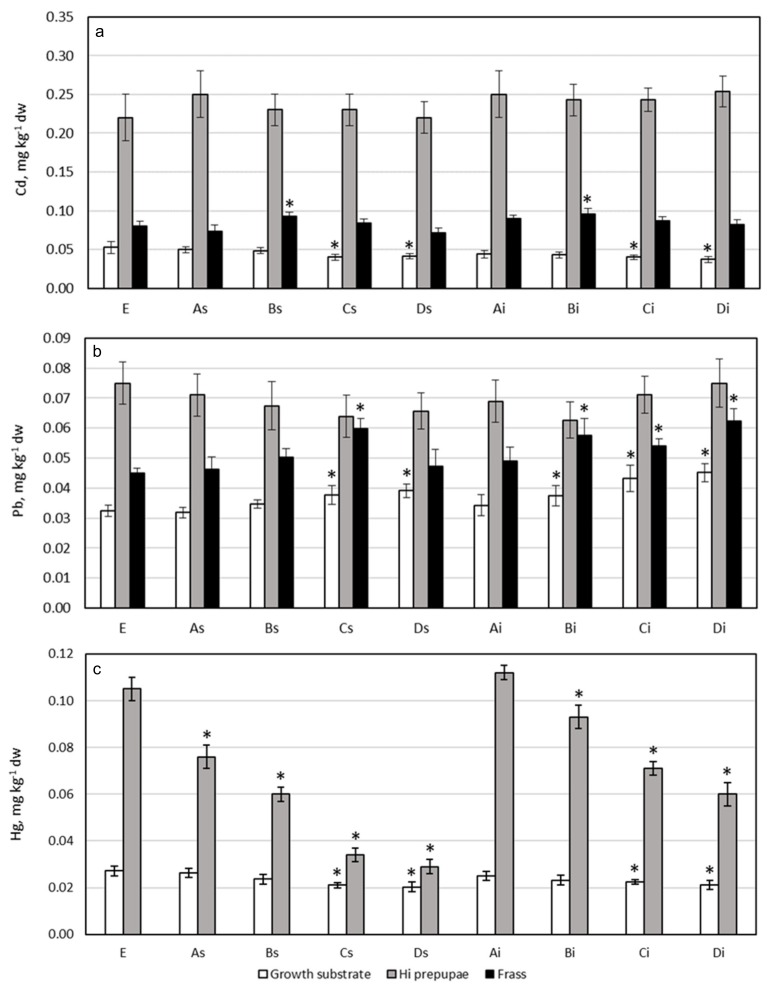
Cd (**a**), Pb (**b**) and Hg (**c**) concentration in growth substrate (white bars), and corresponding HI prepupae (gray bars) and frass (black bars). E: control substrate 100% coffee silverskin; As, Bs, Cs, and Ds: substrates enriched with 5%, 10%, 20%, and 25% of *Schizochytrium* sp., respectively; Ai, Bi, Ci, and Di: substrates enriched with 5%, 10%, 20%, and 25% of *Isochrysis* sp., respectively. Each bar represents the mean ± standard deviation (*n*=9). *: indicates statistically significant differences (*p* < 0.05) within the same matrix with respect to control group E.

**Figure 2:**
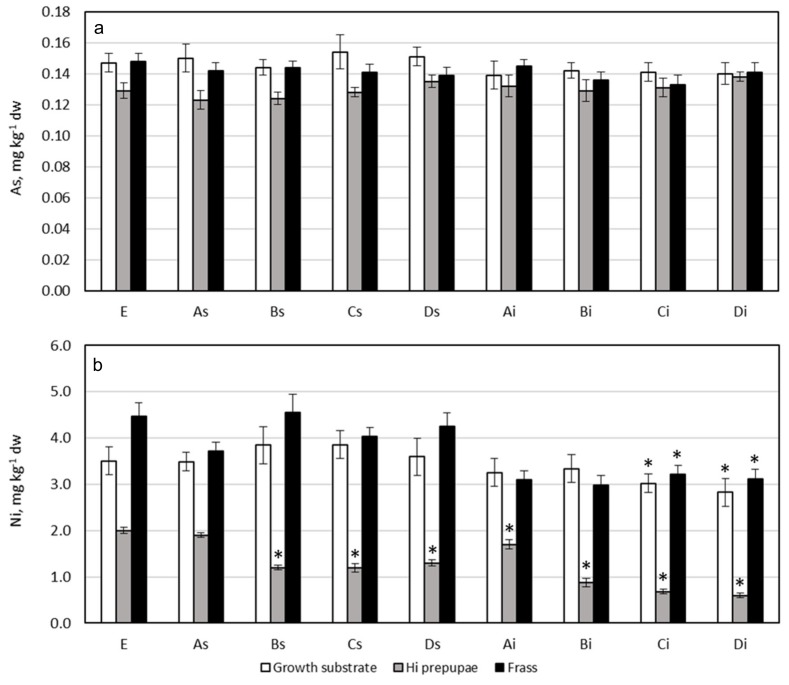
As (**a**) and Ni (**b**) concentration in growth substrate (white bars), and corresponding HI prepupae (gray bars) and frass (black bars). E: control substrate 100% coffee silverskin; As, Bs, Cs, and Ds: substrates enriched with 5%, 10%, 20%, and 25% of *Schizochytrium* sp., respectively; Ai, Bi, Ci, and Di: substrates enriched with 5%, 10%, 20%, and 25% of *Isochrysis* sp., respectively. Each bar represents the mean ± standard deviation (*n* = 9). * indicates statistically significant differences (*p* < 0.05) within the same matrix with respect to control group E.

Figure 1 and Figure 2 show PTEs content in growth substrates, HI prepupae, and frasses. Table 3 shows the bioaccumulation factor (BAF) for prepupae of HI reared on tested growth substrates, calculated on a dry weight basis.

### 3.2. Cadmium

Growth substrate: Considering that Cd content in microalgae was one order of magnitude lower than its concentration in CS (Table 2), the concentration of this metal in the growth substrate was mainly influenced by its content in CS, varying from 0.037 to 0.050 mg kg^−1^ dw (Figure 1). These concentrations are consistent or lower with respect to Cd content recorded in different HI growth substrates, such as plant/macroalgae-based medium [25,32], or cereal processing leftovers [52]. The inclusion of microalgae in the growth substrate led to a reduction in Cd content with respect to the control substrate E, but a statistically significant reduction (*p* = 0.0046) was evidenced only with the inclusion of 20% and 25% of *Schizochytrium* sp. (substrates Cs and Ds, respectively) or *Isochrysis* sp. (substrates Ci and Di, respectively). Referring to the EC limit 2002/32/EC [53] on undesirable substances in animal feed, the legal limit for Cd in feed materials of vegetable origin is 1 mg kg^−1^, in feed materials of animal origin is 2 mg kg^−1^, and for complete feed is 0.5 mg kg^−1^ (maximum content relative to a feeding stuff with a moisture content of 12%). Cd content in tested growth substrates (calculated for a moisture content of 12%) ranged from 0.033 to 0.046 mg kg^−1^ (Table S1), underlying their safety from the point of view of Cd content.

HI prepupae: Cd content in prepupae ranged from 0.19 to 0.24 mg kg^−1^ dw (Figure 1), and no statistically significant differences were evidenced among prepupae reared on different growth substrates (*p* = 0.07). Data are consistent or lower with respect to literature results about HI reared on different natural growth substrates [25,32,52]. Compared with other insect species, the Cd content found in the HI prepupae was higher than that recorded in *Tenebrio molitor* reared on substrates made up of organic wheat milling and olive processing by-products [24], or in edible grasshoppers (*Oxya Chinensis Formosana)* from Korea [54]. Referring to the EC limit 2002/32/EC [53] on undesirable substances in animal feed, HI prepupae showed Cd concentrations within the limit (Table S1).

No statistically significant correlation was found between Cd content in growth substrates and prepupae (*p* = 0.8745). The BAF for Cd was > 1 for all groups (Table 3), with a mean ±SD of 5.6 ± 0.8, indicating that HI prepupae bioaccumulate this metal. Similar BAF values were reported in the literature for HI prepupae reared on different growth substrates, such as 5.8 ± 1.0 [34] and ~2.5 [32] for chicken feed, 5.2 for cereal processing leftovers [52], and ~4.2 for wheat bran [33]. The ability to accumulate Cd is typical of various Dipteran species [55,56], and it is explained by the active transport of this metal by means of heat shock proteins and by the capacity of Cd to pass through Ca^2+^ channels; the hydrated Cd ion has a similar ionic radius to that of the Ca ion, and Cd will therefore be taken up to some extent through Ca-ion pumps [57]. HI larvae have a very high Ca content compared to other insect species [58], and they can accumulate high quantities of Cd, as confirmed by other studies [25,32,33,34,52]. Cd accumulation has been demonstrated also for other insect species, such as *Eligma narcissus*, *Holotrichia*, *Acrida chinensis*, and *Locusta migratoria manilensis* [59], whereas *Tenebrio molitor* did not seem capable to accumulate Cd [24,34]. Cd content depends on the insect life stage: different authors showed a decrease in Cd content passing from HI larvae to prepupae to adults [32,33]. In this context, HI prepupae are preferable to larvae as regards the production of insect biomass for possible application in feed and food production. Cd accumulation in HI larvae and prepupae should be considered carefully, as Cd content in their body increases with increasing Cd concentration in substrates [26,32,34]. Growth substrates used to rear HI insects in this study showed low Cd concentration, and therefore HI prepupae had a Cd content within the legal limit.

Frass: Cd content ranged from 0.071 to 0.096 mg kg^−1^ dw, and a significant higher level of Cd with respect to control group E was found in frasses corresponding to growth substrates with 10% of microalgae (*p* = 0.005) (Figure 1). This is an interesting result, but the authors do not have enough data to speculate on the reason of this result, which should be further investigated. Cd content in frasses was consistently lower than Cd content in HI prepupae, reinforcing the hypothesis that this metal was accumulated in the insect body [34].

### 3.3. Lead

Growth substrate: Lead content in growth substrates ranged from 0.032 to 0.045 mg kg^−1^ dw (Figure 1). A statistically significant increase in Pb content with respect to control group E was evidenced in growth substrates with 10% of *Isochrysis* sp. (Bi) and 20% and 25% of microalgae (i.e., Cs and Ds for *Schizochytrium* sp.; Ci and Di for *Isochrysis* sp.) (*p* < 0.0001). Pb content in the growth substrate was similar or lower than Pb levels found in other substrates, such as chicken pellets [32], by-products of plant processing [52], seaweed-enriched media [25], or in vegetables [60]. Referring to the EC limit 2002/32/EC [53] on undesirable substances in animal feed, the legal limit for Pb in feed materials of animal origin is 10 mg kg^−1^ and for complete feed is 5 mg kg^−1^ (maximum content relative to a feeding stuff with a moisture content of 12%). Pb content in tested growth substrates (calculated for a moisture content of 12%) was in the range of 0.028 to 0.040 mg kg^−1^ (Table S1), well below the legal limit, suggesting that the growth substrates tested in the present study were safe from the point of view of Pb content.

HI prepupae: Pb content in prepupae ranged from 0.063 to 0.075 mg kg^−1^ dw (Figure 1), and no significant differences were evidenced among prepupae reared on different substrates. These Pb levels are consistent with Pb content found in HI prepupae reared on different substrates, such as processed wheat seaweed-enriched growth substrate [25] or corn semolina [20], but are lower than Pb levels found in larvae reared on by-products of plant processing [52]. Compared to other insect species, the Pb content found in HI prepupae was lower than that recorded in *Tenebrio molitor* [24], or in edible grasshoppers (*Oxya Chinensis Formosana)* from Korea [54]. Referring to the EC limit (2002/32/EC) [53] on undesirable substances in animal feed, HI prepupae showed Pb concentrations within the limit (Table S1).

No statistically significant correlation was found for Pb content in growth substrates and prepupae (r = 0.04352, *p* = 0.9115). On the other hand, the BAF value ranged from 1.6 to 2.3, with a mean of 1.9 ± 0.3 (Table 3). This BAF value indicates that HI prepupae bioaccumulate Pb. Various BAFs were reported in literature for HI prepupae reared on different growth substrates, such as 1.2–1.4 for chicken feed [34], 2.7 ± 0.6 for cereal processing leftovers [52], and 2.3 in corn semolina [20], but Diener et al. (2015) [32] reported a BAF value <1. Accumulation of Pb has also been demonstrated in other insect species, such as *T. molitor* [24], grasshoppers [61], and *Spodoptera litura* [62], whereas Pb bioaccumulation was not observed in the locust *L. migratoria* [59]. Moreover, it has been demonstrated that HI larvae exuviae showed higher Pb concentrations than HI larvae or their feed [32], indicating that Pb is sequestered in the exoskeleton of the HI. Thus, Pb content usually decreases during metamorphosis [32]. After all, these studies showed that Pb bioaccumulates in insects, and its degree of bioaccumulation in HI prepupae depends on the initial concentration in the growth substrate and on the life stage.

Frass: Pb content in frasses (from 0.045 to 0.062 mg kg^−1^ dw, Figure 1) was always lower than the corresponding content in HI prepupae, suggesting (as proposed also for Cd) that this metal was accumulated in the body of HI prepupae [34]. A statistically significant increase in Pb content with respect to control group E was evidenced in growth substrates with 20% of *Schizochytrium* sp. (Cs), and with 20% and 25% of *Isochrysis* sp. (Ci and Di) (*p* = 0.0004). A statistically significant correlation was found between Pb content in frass and growth substrates (r = 0.7485, *p* = 0.0203), and the ratio of Pb concentration between frass and the corresponding growth substrate ranged from 0.6 to 0.94, as reported by Purschke et al. (2017) [20]. Therefore, Pb content in frass was influenced by Pb content in the corresponding growth substrate.

### 3.4. Mercury

Growth substrate: Mercury content in the tested growth substrates ranged from 0.020 to 0.027 mg kg^−1^ dw (Figure 1). Similar Hg content was observed from Biancarosa et al. (2018) [25] in processed wheat seaweed-enriched growth substrates. In microalgae there was a significantly lower Hg content with respect to the CS ingredient, and the increase in the percentage inclusion of microalgae to 20% and 25% in growth substrates led to a statistically significant reduction of Hg content (*p* < 0.0001). Referring to the EC limit (2002/32/EC) [53] on undesirable substances in animal feed, the legal limit for Hg in the feed material of animal origin is 0.1 mg kg^−1^ and for complete feed is 0.2 mg kg^−1^ (maximum content relative to a feeding stuff with a moisture content of 12%). Hg content in tested growth substrates (calculated for a moisture content of 12%) was in the range of 0.024 to 0.018 mg kg^−1^ (Table S1), approximately 5- or 10-fold lower than legal limit. Consequently, the tested growth substrates were safe from the point of view of Hg content.

HI prepupae: Hg content in prepupae ranged from 0.029 to 0.112 mg kg^−1^ dw (Figure 1). In general, the microalgae inclusion in growth substrates led to a statistically significant decrease in Hg content in the corresponding HI prepupae with respect to control group E (*p* < 0.0001). These Hg levels are consistent with the Hg content found in HI prepupae reared on corn semolina [20]. Compared with other insect species, Hg content found in HI prepupae was consistent with that found in four species of Thai insects, such as *Patanga succincta*, *Holotrichia* sp., *Acheta domesticus*, and *Bombyx mori* L [63]. Referring to the EC limit (2002/32/EC) [53] on undesirable substances in animal feed, HI prepupae showed Hg concentrations within the legal limit (Table S1).

A statistically significant positive linear correlation was found between Hg content in HI prepupae and corresponding growth substrates (r = 0.8235, *p* = 0.0034), indicating a strong relationship between the variables. The r-squared statistic indicates that the model, as fitted, explains 67.8% of the variability of Hg content in HI prepupae, which was clearly influenced by Hg content in the growth substrate. The BAF value for Hg was > 1 for all groups, indicating that HI prepupae bioaccumulated this metal, as reported in literature for HI [25] and for other insect species, such as *Tenebrio molitor* [24,25,64]. Bioaccumulation and biomagnification of mercury through food webs is well-known [65], and for this reason this metal deserves special monitoring along the feed/food chain. The BAF value was from 4.5 to 1.4, and it decreased with the increase in the microalgae percentage inclusion in the growth substrate. This is an interesting result that should be further investigated.

### 3.5. Arsenic

Growth substrate: As content in the tested growth substrates ranged from 0.139 to 0.154 mg kg^−1^ dw (Figure 2), and no statistically significant differences were found among them (*p* = 0.2345). These levels are of the same order of magnitude than As content found in processed wheat used as diet for HI larvae [24]. Referring to the EC limit (2002/32/EC) [53] on undesirable substances in animal feed, the legal limit for As in feed materials and complete feed is 2 mg kg^−1^ (maximum content relative to a feeding stuff with a moisture content of 12%). Arsenic content in tested growth substrates (calculated for a moisture content of 12%) was in the range of 0.122 to 0.135 mg kg^−1^, approximately 15-fold lower than legal limit (Table S1); the tested growth substrates were safe from the point of view of As content.

HI prepupae: Arsenic content in prepupae ranged from 0.123 to 0.138 mg kg^−1^ dw (Figure 2), similar to As levels found in literature for the same species [25,31] and for other species, such as edible grasshopper (*Oxya Chinensis Formosana)* [54]. HI prepupae reared on substrates containing 25% of microalgae *Schizochytrium* sp. or *Isochrysis* sp. (substrates Ds and Di, respectively) showed higher levels of arsenic than other groups, and this result was well related to the higher content of arsenic in microalgae with respect to CS. In any case, no significant differences were evidenced among groups (*p* = 0.071).

No statistically significant correlation was found between arsenic content in growth substrates and prepupae (r = −0.3460, *p* = 0.3617). Referring to the EC limit (2002/32/EC) [53] on undesirable substances in animal feed, HI prepupae showed As concentrations within the legal limit (Table S1). As content in HI larvae reflected the element content of growth substrates, as BAF value was next to 1, ranging between 0.82 to 0.99 (Table 3). Moreover, As did not accumulate in the body of HI prepupae, as already demonstrated by other studies for black soldier fly and for other species, such as *Tenebrio molitor* [20,24,34]. However, some authors showed that HI larvae accumulate As from the growth substrate [25]. Andrahennadi and Pickering (2008) [66] demonstrated that larvae of Bertha armyworm (*Mamestra configurata* Wlk., Lepidoptera) accumulated arsenic, but that a consistent elimination occurred during larval development, resulting in a drastic reduction of arsenic levels in pupae and adults. The decrease in PTEs during metamorphosis has also been demonstrated for Cd, Pb, Zn, and Cr in HI [32,33]. Therefore, different results about As accumulation in black soldier fly obtained in this study for HI prepupae and in Biancarosa et al. (2018) [25] for HI larvae could be due to a different life stage considered. This interesting topic deserves further investigation. Previous studies demonstrated that the level of As in invertebrates depends more strongly on taxonomical differences than on exposure level [67]. In particular, Odonata and Lepidoptera showed higher levels than Diptera and Orthoptera from the same location. The low As content found in HI prepupae in this study seems consistent with pattern described for different orders.

Frass: The arsenic content in frasses (from 0.133 to 0.145 mg kg^−1^ dw, see Figure 2) was generally higher than the corresponding levels in prepupae, confirming that this element was not accumulated by HI.

### 3.6. Nickel

Growth substrate: The addition of different percentages of *Schizochytrium* sp. to CS did not modify Ni content in growth substrates because of the similar concentration of this element in these two ingredients. Conversely, the statistically significant lower Ni content of *Isochrysis* sp. with respect to CS caused a decrease in Ni content in growth substrates in relation to the increase of *Isochrysis* sp. inclusion. In particular, a significantly lower content of Ni in growth substrates with the inclusion of 20% (Ci) and 25% (Di) *Isochrysis* sp. was evidenced with respect to control growth substrate E and substrates with the inclusion of *Schizochytrium* sp. (As-Ds) (*p* = 0.009) (Figure 2). No legal limits are presently reported for Ni in feed and food.

HI prepupae: Ni content in HI prepupae ranged from 0.60 to 2.0 mg kg^−1^ dw, and no significant correlation was found between Ni content in the growth substrates and prepupae (r = 0.4498, *p* = 0.2245). Based on Boyd (2009) [68], they can be classified as low-Ni insect, having a Ni content < 500 mg kg^−1^ dw. However, significant differences were evidenced among HI prepupae reared on different substrates (*p* < 0.0001) (Figure 2). HI prepupae reared on control substrate E and on substrates including 5% of microalgae *Schizochytrium* sp. (As) or *Isochrysis* sp. (Ai) showed the highest Ni concentration, which significantly decreased with the increasing percentage of microalgae inclusion. HI prepupae reared on substrates containing 10%, 20%, and 25% of *Isochrysis* sp. showed the lowest concentrations of this metal. If the decrease in Ni content in HI prepupae reared on *Isochrysis* sp.-enriched substrates can be explained by the Ni decrease in the corresponding growth substrates, the same did not happen for prepupae reared on *Schizochytrium* sp.-enriched substrates. In this case, although Ni content did not vary significantly in tested substrates, HI prepupae reared on substrates with 10%, 20%, and 25% inclusion of microalgae showed a significant lower Ni content with respect to substrates E and As. A different bioavailability of this metal between ingredients of the growth substrates can be supposed. The results on BAF support this hypothesis: the BAF for Ni was < 1 for all groups (Table 3), demonstrating that Ni did not bioaccumulate, but the BAF of HI prepupae reared on control substrate E and on substrates including 5% of microalgae *Schizochytrium* sp. (As) or *Isochrysis* sp. (Ai) showed a BAF with a mean ±SD of 0.55 ± 0.02, approximately 2-fold higher than BAF value of HI prepupae reared on substrates containing 10%, 20%, and 25% of microalgae (from 0.21 to 0.36, mean 0.24 ± 0.06). This last BAF value was consistent with that of HI larvae reared on corn-based substrates [20]. Evidently, Ni uptake of HI depends on the growth substrate, and this result should be further investigated. The BAF <1 for Ni agrees with the observations of Sun et al. (2007) [69]: they demonstrated that pupae and adults of *Spodoptera litura* did not accumulate Ni. Moreover, they observed that Ni concentrations in pupae and adults were significantly lower than those in larvae, which indicated that the excessive nickel might be excreted during metamorphosis, as observed also for the other PTEs indagated.

Frass: The Ni content in frasses varied in the range of 3.0 to 4.6 mg kg^−1^ dw; frasses deriving from growth substrates enriched with 20% and 25% of *Isochrysis* sp. showed a significant lower Ni content with respect to control substrates and growth substrates *Schizochytrium* sp.-enriched (*p* < 0.0001). Ni content in frasses was always higher than Ni content in the corresponding prepupae, as also evidenced in the literature for HI larvae reared on corn-based substrates [20]. Therefore, in agreement with the BAF lower than 1, we can affirm that this element is not accumulated by this insect species, suggesting that Ni present in the growth substrate penetrates in the body of prepupae and was then excreted without bioaccumulation. To further support this hypothesis, a significant correlation has been demonstrated between Ni content in frasses and growth substrates (r = 0.7834, *p* = 0.0125).

### 3.7. Potentially Toxic Elements Content in HI Prepupae and Comparison with Legal Limit for Food

In Table 4 we reported Cd, Pb, Hg, As, and Ni content in HI prepupae, referred to wet weight (ww), to make a comparison with their legal limit for food (EU commission regulation No 1881/2006 of 19 December, 2006, setting maximum levels for certain contaminants in foodstuffs, and amending Regulations No 420/2011 of 29 April, 2011, and No 1006/2015, of 25 June, 2015 as regards maximum levels of inorganic arsenic in foodstuffs) [38]. Cd showed a mean concentration in HI prepupae of 0.076 ± 0.004 mg kg^−1^ ww, higher than Cd content found in mealworm larvae of *T. molitor* [24], in edible grasshoppers (*Oxya Chinensis Formosana)* [54], or in others edible insects, such as *Galleria mellonella*, *Locusta migratoria*, or *Alphitobius diaperinu*) [70]. Cd content found in HI larvae was high, but in any case, was lower than the legal limit of 0.20 referred to meat. Pb showed a mean content of 0.021 ± 0.002 mg kg^−1^ ww, 4–5-fold lower than the legal limit for food (referred to meat). Compared with other insect species, Pb content found in HI prepupae was lower than that recorded in *Tenebrio molitor* reared on substrates made up of organic wheat milling and olive processing by-products [24], or in edible grasshoppers (*Oxya Chinensis Formosana)* from Korea [54], but higher than Pb content recorded in other various edible insects [70]. Hg mean content was 0.022 ± 0.008, 15–50-fold lower than the legal limit referred to Hg in fish fillet. Hg content is consistent with that found in other insect species, such as wild Thai insects, *Patanga succincta*, *Holotrichia* sp., *Acheta domesticus*, and *Bombyx mori* L [63]. As showed a mean content of 0.041 ± 0.004 mg kg^−1^ ww, 4–5-fold lower then legal limit for As in rice, husked rice, milled rice, and parboiled rice, as defined in Codex Standard 198-1995 (Commission regulation (EU) 2015/1006 of 25 June 2015). Similar results were obtained for the same specie [25,31] and for other species, such as edible grasshopper (*Oxya Chinensis Formosana)* [54]. No legal limits were reported for Ni in food. From our results, we observed an accumulation of Cd, Pb, and Hg in HI prepupae. However, the concentrations of indagated PTEs did not exceed the legal limit. Then, it seems that HI prepupae reared on coffee by-products and microalgae represent a chemically safe way to produce insect biomass for possible application in food production.

## 4. Conclusions

The interest about chemical hazards associated with insects as potential ingredients for feed and food is increasing worldwide, and this study adds important information, evaluating a possible practical use of HI prepupae reared on coffee by-products and microalgae.

HI prepupae accumulated Cd, Pb, and Hg from growth substrates based on coffee roasted by-product and microalgae. This observation underlines that safe HI production as ingredient for feed or food, needs a strict control of these undesirable contaminants both in the initial substrate as well as in the final product (HI prepupae and their products). However, the content of PTEs in HI prepupae was always lower than the legal limit both for feed and food, even if Cd content was next to the legal limit for food. As was not accumulated by HI prepupae, in accordance with the low capacity of Diptera to accumulate this metalloid. Consequently, As content is lower than the legal limit both for feed and food.

Overall, our results indicate that the risk of exposure to PTEs when using HI prepupae reared on growth substrates based on CS and microalgae as ingredient for feed and food is relatively low and in compliance with European Union regulations.

However, it should be pointed out that Ni deserves a separate discussion, as at present, Ni is not considered in the laws that limit PTEs content in feed and food and only a few studies on its accumulation in insects are available. Considering the level of this metal detected in HI prepupae, and considering its toxicity, the authors think that it would be interesting to deepen this topic and suggest the scientific community to identify and fix specific limits for Ni content both in feed and food.

As a final remark, as the content of PTEs in HI prepupae depends on the growth substrate, the authors suggest to layout, in addition to a list of insect species which may be used for the production of processed animal protein, a specific list of tested growth substrates to be used in a safe way for edible-insects production.

## Figures and Tables

**Table 1:** Accuracy test using certified reference material DORM-2 (dog fish muscle), NRC Canada. Data are expressed in mg kg^−1^.

Element	Analytical Method	Analytical Result (*n* = 9)	Certified Value	Δ (%)
Cd	GF-AAS	0.042 ± 0.005	0.043 ± 0.008	−2
Pb	GF-AAS	0.068 ± 0.003	0.065 ± 0.007	+5
As	GF-AAS	17.4 ± 0.6	18 ± 1.1	−3
Ni	GF-AAS	18 ± 1.2	19.4 ± 3.1	−7
Hg	DMA-1	4.21 ± 0.06	4.58 ± 0.16	−8

**Table 2:** PTEs content (mg kg^−1^ dw) in ingredients used to prepare growth substrates.

Ingredients	Cd	Pb	Hg	As	Ni
Silverskin	0.053 ± 0.008 ^b^	0.032 ± 0.002 ^a^	0.027 ± 0.001 ^c^	0.147 ± 0.006 ^a^	3.5 ± 0.2 ^b^
*Schizochytrium* sp.	0.0025 ± 0.0002 ^a^	0.065 ± 0.003 ^b^	0.009 ± 0.002 ^b^	0.184 ± 0.002 ^b^	3.6 ± 0.1 ^b^
*Isochrysis* sp.	0.0020 ± 0.0005 ^a^	0.084 ± 0.009 ^c^	0.0016 ± 0.0004 ^a^	0.153 ± 0.001 ^a^	1.18 ± 0.03 ^a^
*p*-value	<0.0001	0.0002	<0.0001	<0.0001	<0.0001

Means within columns bearing different letters indicate statistically significant differences among ingredients (*p* value reported in the table for each element).

**Table 3:** Bioaccumulation factor (BAF) of prepupae of *Hermetia illucens* reared on tested growth substrates, calculated on a dry weight basis.

HI Prepupae	Cd	Pb	Hg	As	Ni
HI E	4.2 ± 0.9	2.3 ± 0.3	3.9 ± 0.2	0.88 ± 0.05	0.57 ± 0.05
HI As	5.0 ± 0.7	2.2 ± 0.3	2.9 ± 0.2	0.82 ± 0.05	0.54 ± 0.03
HI Bs	4.8 ± 0.5	1.9 ± 0.2	2.5 ± 0.1	0.86 ± 0.03	0.31 ± 0.03
HI Cs	5.7 ± 0.8	1.7 ± 0.2	1.6 ± 0.1	0.83 ± 0.06	0.31 ± 0.03
HI Ds	5.3 ± 0.7	1.7 ± 0.2	1.4 ± 0.1	0.89 ± 0.03	0.36 ± 0.04
HI Ai	5.7 ± 0.9	2.0 ± 0.3	4.5 ± 0.2	0.95 ± 0.08	0.52 ± 0.06
HI Bi	5.6 ± 0.7	1.7 ± 0.2	4.0 ± 0.2	0.91 ± 0.06	0.26 ± 0.04
HI Ci	6.1 ± 0.6	1.6 ± 0.2	3.2 ± 0.1	0.93 ± 0.06	0.23 ± 0.02
HI Di	6.9 ± 0.9	1.7 ± 0.2	2.8 ± 0.1	0.99 ± 0.05	0.21 ± 0.03

HI E: prepupae reared on control substrate E (100% coffee silverskin, CS); HI As, HI Bs, HI Cs, and HI Ds: prepupae reared on substrate CS enriched with 5%, 10%, 20%, and 25% of *Schyzochytrium* sp., respectively; HI Ai, HI Bi, HI Ci, and HI Di: prepupae reared on substrate CS enriched with 5%, 10%, 20%, and 25% of *Isochrysis* sp., respectively. Data represent mean ± standard deviation (*n* = 9).

**Table 4:** Cadmium (Cd), lead (Pb), mercury (Hg), arsenic (As), and nickel (Ni) concentrations (mg kg^−1^ ww) in HI prepupae, and legal limits for food (Directive 1881/2006/EU and amending regulations 420/2011/EU and 1006/2015/EU).

HI Prepupae	Cd	Pb	Hg	As	Ni
Legal limit	0.050-0.20 ^a^ (meat)	0.10 ^a^ (meat)	0.50 ^a^ (fish fillet)	0.20 ^b^ (rice)	-
HI E	0.076 ± 0.010	0.026 ± 0.002	0.030 ± 0.001	0.044 ± 0.002	0.76 ± 0.02
HI As	0.072 ± 0.009	0.021 ± 0.002	0.024 ± 0.002	0.036 ± 0.002	0.54 ± 0.01
HI Bs	0.072 ± 0.006	0.021 ± 0.002	0.020 ± 0.001	0.039 ± 0.001	0.36 ± 0.02
HI Cs	0.078 ± 0.007	0.022 ± 0.002	0.012 ± 0.001	0.043 ± 0.001	0.39 ± 0.03
HI Ds	0.076 ± 0.007	0.023 ± 0.002	0.010 ± 0.001	0.047 ± 0.001	0.46 ± 0.02
HI Ai	0.074 ± 0.005	0.020 ± 0.002	0.032 ± 0.003	0.037 ± 0.002	0.49 ± 0.03
HI Bi	0.076 ± 0.008	0.018 ± 0.002	0.029 ± 0.005	0.040 ± 0.002	0.27 ± 0.03
HI Ci	0.084 ± 0.006	0.020 ± 0.002	0.024 ± 0.003	0.045 ± 0.002	0.24 ± 0.02
HI Di	0.076 ± 0.006	0.021 ± 0.002	0.018 ± 0.005	0.041 ± 0.001	0.18 ± 0.01

^a^ 1881/2006/EU and 420/2011/EU. ^b^ 1006/2015/EU. HI E: prepupae reared on substrate E (100% coffee silverskin, CS); HI As, HI Bs, HI Cs, and HI Ds: prepupae reared on substrate CS enriched with 5%, 10%, 20%, and 25% of *Schyzochytrium* sp., respectively; HI Ai, HI Bi, HI Ci, and HI Di: prepupae reared on substrate CS enriched with 5%, 10%, 20%, and 25% of *Isochrysis* sp., respectively. Data represent mean ± standard deviation (*n* = 9).

## Supplementary Materials

The following are available online at https://www.mdpi.com/1660-4601/17/6/2121/s1, Table S1: Concentration (mg kg^−1^) of cadmium (Cd), Lead (Pb), mercury (Hg), Arsenic (As), and nickel (Ni) converted to moisture content of 12%, in the growth substrates and in HI prepupae. Legal limits for feed material and complete feed (relative to a moisture of 12%), according to Directive 2002/32/EU (and amendments) on undesirable substances in animal feed.
