# Supplementary material for: A Chemically Safe Way to Produce Insect Biomass for Possible Application in Feed and Food Production
Source: Int J Environ Res Public Health. 2020 Mar 23;17(6):2121. doi: 10.3390/ijerph17062121 (PMC7142791; doi:10.3390/ijerph17062121)
Supplement: Supplementary file 1 [file ijerph-17-02121-s001.pdf]

A chemically safe way to produce insect biomass for possible application in feed and food production

Cristina Truzzi[1*], Anna Annibaldi[1*], Federico Girolametti[1], Leonardo Giovannini[1], Paola Riolo[2], Sara Ruschioni[2], Ike Olivotto[1], Silvia Illuminati[1]

[1] Dipartimento di Scienze della Vita e dell'Ambiente, Università Politecnica delle Marche, via Brecce Bianche 60131, Ancona, Italy
[2] Dipartimento di Scienze Agrarie, Alimentari ed Ambientali, Università Politecnica delle Marche, via Brecce Bianche 60131, Ancona, Italy
[*] Correspondence: c.truzzi@univpm.it (C.T.); a.annibaldi@univpm.it (A.A.)

Supplementary Material

Table S1 Concentration (mg kg^{-1}) of cadmium (Cd), Lead (Pb), mercury (Hg), Arsenic (As) and nickel (Ni) converted to moisture content of 12%, in the growth substrates and in HI prepupae. Legal limits for feed material and complete feed (relative to a moisture of 12%), according to Directive 2002/32/EU (and amendments) on undesirable substances in animal feed.

Samples	Cd	Pb	Hg	As	Ni
Legal limit					
Complete feed	0.5	5.0	0.2	2.0	-
Feed materials	1 (vegetable)<br>2 (animal)	10	0.1	2.0	-
Schizochytrium	0.0022±0.0001	0.057±0.003	0.008±0.002	0.162±0.002	3.2±0.1
Isochrysis	0.001±0.001	0.073±0.009	0.0014±0.004	0.135±0.001	1.04±0.03
E	0.046±0.007	0.028±0.002	0.024±0.002	0.129±0.005	3.1±0.3
As	0.044±0.003	0.028±0.002	0.023±0.002	0.132±0.008	3.1±0.2
Bs	0.043±0.003	0.030±0.001	0.021±0.002	0.127±0.004	3.4±0.4
Cs	0.035±0.003	0.033±0.003	0.018±0.001	0.135±0.01	3.4±0.3
Ds	0.036±0.003	0.034±0.002	0.018±0.002	0.133±0.005	3.2±0.4
HI E	0.19±0.03	0.066±0.006	0.092±0.004	0.114±0.004	1.94±0.06
HI As	0.24±0.03	0.062±0.006	0.050±0.004	0.108±0.002	1.65±0.04
HI Bs	0.20±0.02	0.059±0.007	0.040±0.003	0.109±0.003	1.03±0.04
HI Cs	0.20±0.02	0.056±0.006	0.022±0.003	0.113±0.003	1.02±0.08
HI Ds	0.19±0.02	0.058±0.005	0.019±0.003	0.119±0.002	1.18±0.06
Ai	0.039±0.004	0.030±0.002	0.022±0.002	0.122±0.008	2.6±0.3
Bi	0.038±0.004	0.033±0.003	0.020±0.002	0.125±0.004	2.9±0.3
Ci	0.035±0.003	0.038±0.003	0.020±0.001	0.124±0.005	2.7±0.2
Di	0.033±0.004	0.040±0.001	0.019±0.002	0.123±0.006	2.5±0.3
HI Ai	0.24±0.03	0.061±0.006	0.099±0.003	0.116±0.008	1.52±0.09
HI Bi	0.21±0.02	0.055±0.005	0.078±0.004	0.114±0.008	0.77±0.08
HI Ci	0.21±0.01	0.063±0.005	0.063±0.003	0.115±0.006	0.60±0.04
HI Di	0.22±0.02	0.066±0.007	0.040±0.004	0.122±0.002	0.52±0.04

Substrates E: 100% coffee silverskin (CS); substrates As, Bs, Cs and Ds: CS enriched with 5%, 10%, 20% and 25% of *Schyzochytrium* sp., respectively; substrates Ai, Bi, Ci and Di: CS enriched with 5%, 10%, 20% and 25% *Isochrysis* sp., respectively. HI E: prepupae reared on substrate E; HI As, HI Bs, HI Cs, HI Ds: prepupae reared on As, Bs, Cs and Ds, respectively; HI Ai, HI Bi, HI Ci, HI Di: prepupae reared on substrate Ai, Bi, Ci and Di, respectively. Data represent mean ± standard deviation (n=9)
